# Expressions of candidate molecules in the human fallopian tube and chorionic villi of tubal pregnancy exposed to levonorgestrel emergency contraception

**DOI:** 10.1186/1477-7827-11-46

**Published:** 2013-05-20

**Authors:** Changxiao Huang, Mei Zhang, Chunxia Meng, Wei Shi, Lulu Sun, Jian Zhang

**Affiliations:** 1 Department of Obstetrics and Gynaecology, International Peace Maternity and Child Health Hospital/School of Medicine, Shanghai Jiao Tong University, Shanghai, China; 2 Department of Pathology, Key Laboratory of Cell Differentiation and Apoptosis of Chinese Ministry of Education, Institutes of Medical Sciences, Shanghai Jiao Tong University School of Medicine, Shanghai, China; 3 Department of Medical Pharmacology and Physiology, School of Medicine, University of Missouri-Columbia, Columbia MO, USA

**Keywords:** Tubal pregnancy, Levonorgestrel, Emergency contraception, Fallopian tube, Chorionic villi

## Abstract

**Background:**

Cases of ectopic pregnancy (EP) following levonorgestrel (LNG) emergency contraception (EC) failure were reported, however, the effects of LNG on tubal microenvironment or chorionic villi in EP have not yet been documented.

**Methods:**

Fifty-five women with tubal pregnancy were divided into two groups according to whether LNG-EC was administrated during the cycle of conception. The serum concentrations of beta-hCG, E2 and P were measured. The mRNA and protein expressions of estrogen and progesterone receptors, leukemia inhibitory factor, vascular endothelial growth factor, inducible nitric oxide synthase, and endocannabinoid receptor - CB1 in the ectopic implantation site and chorionic villi were examined.

**Results:**

Compared to those unexposed to LNG-EC, women with tubal pregnancy exposed to LNG-EC during the cycle of conception had no statistically significances in the serum concentrations of beta-hCG, E2 P, nor in the pathological types of tubal pregnancy or the expressions of ER-alpha, PR, LIF, VEGF, iNOS and CB1.

**Conclusions:**

The expressions of candidate molecules in the fallopian tube and chorionic villi were not altered by exposure to LNG-EC. A routine therapy with no additional intervention might thus be applied to tubal pregnancy exposed to LNG-EC.

## Background

Ectopic pregnancy (EP) is the leading cause of pregnancy-related death in the first trimester of pregnancy, and it accounts for approximately 2% of all recognized pregnancies [1]. More than 98% of the EPs occur in the fallopian tube, particularly in the ampulla [2,3]. The pathogenesis of tubal pregnancy remains unclear. One hypothesis is conditions that delay or prevent passage of the embryo through the tube and into the uterine cavity may lead to ectopic implantation [3]. It has been proposed that an elevated progesterone concentration could theoretically impair the fallopian tube cilia motility and thus increase the risk of EP in women using a progestin-only pill [4]. Levonorgestrel (LNG), a synthetic progestogen, is effective and widely used for emergency contraception (EC) either as a single dose of 1.5 mg or in two doses of 0.75 mg with 12 hours apart [5]. When administered within 72 h after an unprotected intercourse, it prevents about 57–93% of unwanted pregnancies resulting from unprotected intercourse [6,7]. The LNG only EC regimen acts via inhibiting or delaying ovulation, but has no detectable effect on the endometrial receptivity or steroid hormone receptors expressed in the fallopian tube from non-pregnant women seeking sterilization [8-10]. Although there is some epidemiological and pharmacological evidence, an eventual causative effect of LNG-EC in the development of EP has not been elucidated. Moreover, compared to intrauterine pregnancy, some changes in the endosalpinx environment and chorionic villi are specific to tubal pregnancy due to the anatomic structures involved. A variety of molecules expressed in the chorionic villi and fallopian tube, such as E2 receptors (ERs), P receptors (PRs), leukemia inhibitory factor (LIF), vascular endothelial growth factor (VEGF), inducible nitric oxide synthase (iNOS), and endocannabinoid receptor 1 (CB1), are potential regulators of the pathophysiological changes in tubal pregnancy [11-25]. Cases of EP following LNG-EC failure were constantly reported as LNG-EC pills are more and more widely used, however, up to now the effect of LNG on the tubal microenvironment or chorionic villi in EP, which is the aim of this study, has not been documented.

## Methods

### Patients and samples

The study was approved by the local ethics committee at International Peace Maternity and Child Health Hospital, Shanghai, China. Written informed consent was obtained from all the women before being recruited and sample collection. During November 2010 and June 2011, women diagnosed with tubal pregnancy exposed and comparable women unexposed to LNG-EC during the cycle of conception were recruited as the LNG-EC group and control group, respectively. None of the women had used any other hormonal contraceptive for a minimum of 3 months before being recruited except the self-administered LNG-EC during the cycle of conception. None of them had acute hemorrhagic shock associated with the tubal pregnancy when recruited. Peripheral blood was collected when they were recruited and then centrifuged. The serum concentrations of beta-hCG, E2 and P were measured using the chemiluminescence immunoassays, which are based on a sandwich antibody principle (Cobas® 6000, Roche, Basel, Switzerland). All of the women were treated with laparoscopic examination followed by salpingectomy. The volume of abdominal hemorrhage was assessed during the surgery. The chorionic villi were separated from the ectopic implantation site in the fallopian tube during surgery. Both isolated chorionic villi and ectopic implantation site tissues in the fallopian tube were collected from each woman. The tissue samples were fixed in 10% neutral buffered formaldehyde followed by dehydration through upgraded series of ethanol and embedding in paraffin.

### Quantitative real-time polymerase chain reaction (qPCR)

Following the tissue homogenization, total RNA was extracted using the TRIzol reagent (Roche, Basel, Switzerland) according to the manufacturer’s instructions. Complementary deoxyribonucleic acid (cDNA) was synthesized from 1000 ng of total RNA in a 20 μl reaction volume (Thermo Scientific Fermentas, MA, USA). The levels of mRNA encoding ER-alpha, PR, LIF, VEGF, iNOS and CB1 were quantified by real-time PCR running on a 7500 Real-time PCR System (Applied Biosystems, CA, USA). Primers were made by Sangon Biotech (Shanghai, China) and the sequences are shown in Table 1. The reaction mixtures in a 10 μl volume contained 5 μl of SYBR Green (Thermo Scientific Fermentas, MA, USA), 0.15 μl mixture of the forward and reverse primers (1 uM for each), 1 μl of cDNA, and 3.85 μl of RNase-free water. The real-time PCR amplification was performed in duplicate using 96-well optical PCR plates. The reaction conditions were 20 seconds at 50°C of pre-treatment, 10 minutes at 95°C of initial denaturation followed by 40 cycles of denaturation for 15 seconds at 95°C and annealing/extension for 60 seconds at 60°C. Threshold cycles (Cts) were determined and the relative quantity of the gene expression was calculated by formula 2^–ΔΔCT^ with glyceraldehyde-3-phosphate dehydrogenase (GAPDH) served as an endogenous control.

**Table 1:** Primer sequences

**Primer**	**Sequences**
GAPDH	(Forward) 5′- ATGGAAATCCCATCACCATCTT-3′
	(Reverse) 5′- CGCCCCACTTGATTTTGG-3′
ER-alpha	(Forward) 5′- GCCAAATTGTGTTTGATGGATTAA-3′
	(Reverse) 5′- CGACAAAACCGAGTCACATCAG-3′
PR	(Forward) 5′- CAGTGGGCGTTCCAAATGA-3′
	(Reverse) 5′- AGCCAAGCCCTAAGCCAGAGATTCACTTT-3′
LIF	(Forward) 5′- GTGTTGCAGGTGCAGCAATT-3′
	(Reverse) 5′- CATCCTCTGCCCACCCT-3′
VEGF	(Forward) 5′-CGAAGTGAAGTTCATGGATG-3′
	(Reverse) 5′-TGTATCAGTCTTTCCTGGT-3′
iNOS	(Forward) 5′-GCGGTAACAAAGGAGATAGAAACAA-3′
	(Reverse) 5′-TGCTTGGTGGCGAAGATGA -3′
CB1	(Forward) 5′- TTCTTCTTACACCCCGGTCTCA-3′
	(Reverse) 5′- TACTGGAAAAAGGCCCAACAAG-3′

ER-alpha: Estrogen receptor-alpha; *PR*, Progesterone receptor; *LIF*, Leukemia inhibitory factor; *VEGF*, Vascular endothelial growth factor; *iNOS*, Inducible nitric oxide synthase; *CB1*, Endocannabinoid receptor 1.

### Immunohistochemistry

For immunostaining of ER-alpha, PR, LIF, VEGF, CB1 and iNOS, the tissue sections were deparaffinized and then rehydrated. Antigen retrieval was appropriately applied (see Table 2). All the sections were subjected to 3% hydrogen peroxide (H_2_O_2_) in methanol for 10 min at room temperature. The sections were then incubated with the primary antibodies overnight in a humidified chamber at 4°C. The sections were subsequently incubated with biotinylated secondary antibodies followed by horseradish peroxidase complex (Sun Biotech, Shanghai, China). The 3, 3′-diaminobenzidine (DAB) substrate kit (ZSGB-BIO, Beijing, China) was applied to visualize the immunostaining, and the sections were then counterstained with hematoxylin. Rinse with tris-buffered saline was performed between each step. Endometrium was used as positive control and negative control was subjected to the identical procedures except incubation with primary antibodies. All immunohistochemical images were evaluated using light microscopy. These photographs were captured at 400 × magnification by a video camera (AxioCam, Zeiss, Germany) and digitized for computerized image analyses. Five fields of a whole section were randomly chosen to avoid errors resulting from uneven staining. The staining was quantitatively analyzed using an Axioplan 2 Imaging Analysis System (KS400 Version 3.0, Zeiss, Germany) according to its protocols [26,27]. The analysis system automatically generated a result data for each field, which was expressed as a ratio of the number of positively-stained cells to the number of all cells. An experienced observer who was blinded to the identity of the slides finally validated the immunostaining results assessed by the computerized image analyses.

**Table 2:** Antibody used for immunohistochemistry

**Name**	**Source**	**Concentration**	**Species**	**Unmasking buffer**	**Antigen retrieval method**
**ER- alpha**	ZSGB-BIO, Beijing, China	1:20	Rabbit monoclonal	EDTA solution	microwave heating on high for 20 minutes
**PR**	ZSGB-BIO, Beijing, China	1:10	Rabbit monoclonal	EDTA solution	microwave heating on high for 20 minutes
**LIF**	Boster, Wuhan, China	1:100	Rabbit monoclonal	citrate buffer	microwave heating on high for 20 minutes
**VEGF**	ZSGB-BIO, Beijing, China	1:150	Rabbit monoclonal	citrate buffer	microwave heating on high for 20 minutes
**CB1**	Enzo Life Sciences International USA	1:150	From rabbit	EDTA solution	microwave heating on high for 20 minutes
**iNOS**	Boster, Wuhan, China	1:200	Rabbit monoclonal	trypsin	induced at room temperature

*ER-alpha*, Estrogen receptor-alpha; *PR*, Progesterone receptor; *LIF*, Leukemia inhibitory factor; *VEGF*, Vascular endothelial growth factor; *iNOS*, Inducible nitric oxide synthase; *CB1*, Endocannabinoid receptor 1.

### Statistical analysis

Comparison of the immunostaining scores between the two groups was performed using the *t*-test by employing the GraphPad Prism software (version 5.0; CA, USA). The data from the quantitative real-time PCR were log transformed and then the unpaired *t*-test was applied for statistical analysis. Chi-square test was used to compare the number of ruptured tubal pregnancy in women between the two groups by employing Statistical Analysis System (SAS) (version 6.12, Raleigh SAS Software Co., NC, USA). All the quantitative data were presented as MEAN +/- SE. A P-value of < 0.05 was considered statistically significant.

## Results

Fifty-five tissue samples were collected from 27 women in the LNG-EC group and 28 women in the control group. Three women were diagnosed ruptured tubal pregnancy by pathologic evaluation, one of whom claimed having administered LNG-EC during the cycle of conception whereas the rest two had not. There were no significant differences in the age, gestational week, volume of abdominal hemorrhage, serum concentrations of beta-hCG, E2, P, or the occurrence of ruptured tubal pregnancy in women between the two groups (Table 3).

**Table 3:** Clinical information on women with tubal pregnancy

**Group**	**N**	**Age**	**Gestation length (d)**	**Abdominal hemorrhage (ml)**	**Serum**			**Rupture of tubal pregnancy**
					**hCG**	**Estadiol**	**Progesterone**	
					**(IU/L)**	**(pmol/L)**	**(nmol/L)**	
LNG-EC	27	28.41+/-0.82	47.74+/-1.80	145.87+/-110.36	5950.2+/-2636.4	866.9+/-124.61	33.86+/-5.72	1
Control	28	32.50+/-1.10	49.14+/-2.11	151.36+/-124.04	5782.1+/-1795.8	1043.5+/-336.36	38.31+/-4.31	2
Total	55							

Values represent mean +/- standard error.

No significant differences were noted between the two groups with respect to age, gestational age, abdominal hemorrhage, serum level of beta-hCG, E2, and P, and the occurrence of tubal pregnancy rupture.

*LNG*, Levonorgestrel; *EC,* Emergency contraception.

### Quantitative real-time PCR

**Figure 1:**
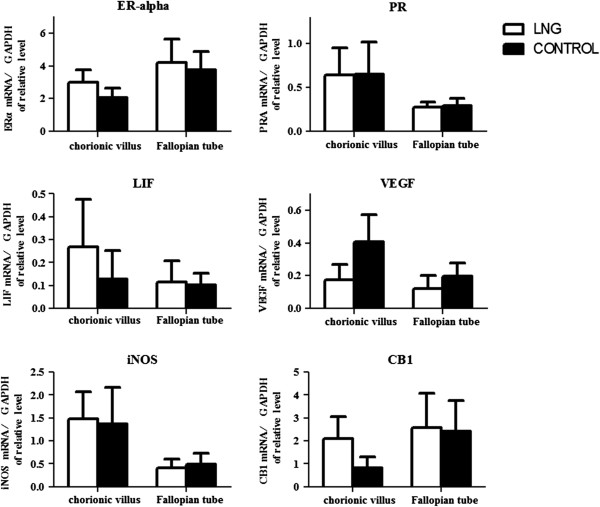
**Quantitative real-time PCR analysis of mRNA expression in chorionic villi and the implantation site in fallopian tube of tubal pregnancy.** There were no significant differences between the two groups in the mRNA expressions of estrogen receptor (ER)-alpha, progestogen receptor (PR), leukemia inhibitory factor (LIF), vascular endothelial growth factor (VEGF), inducible nitric oxide synthase (iNOS), and endocannabinoid receptor 1 (CB1) in chorionic villi and the implantation site in fallopian tube of tubal pregnancy. All mRNA expression values were normalized against the glyceraldehyde-3-phosphate dehydrogenase (GAPDH) endogenous control gene.

There were no significant differences between the two groups in the mRNA expressions of ER-alpha, PR, LIF, VEGF, iNOS, or CB1 expressed either in the chorionic villi or at the implantation site in the fallopian tube (Figure 1).

### Immunohistochemistry

#### Estrogen receptor-alpha

**Figure 2:**
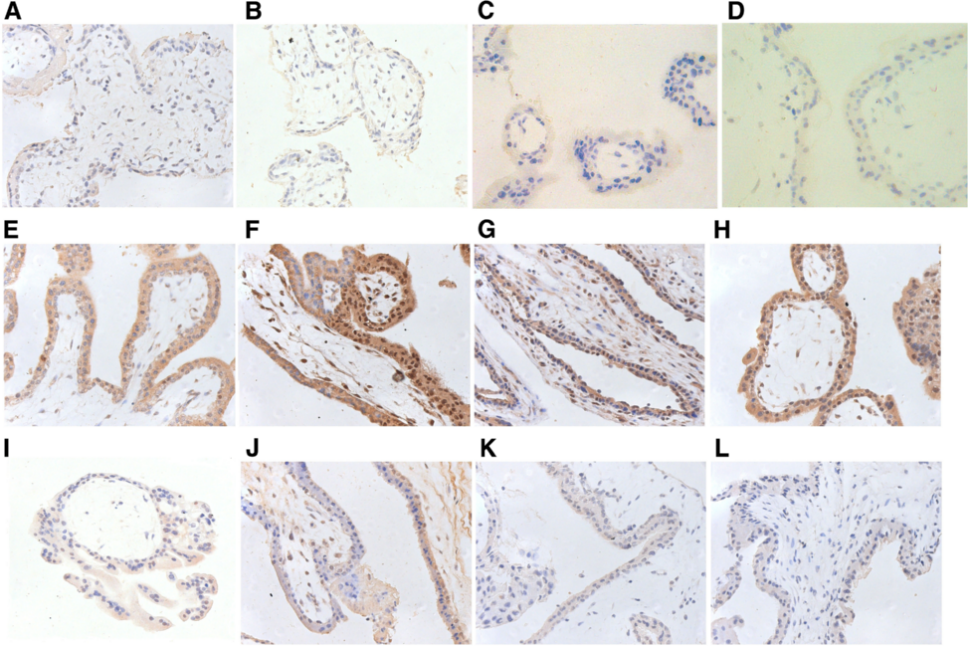
**Immunostaining of the chorionic villi in tubal pregnancy.** Expressions of estrogen receptor (ER)-alpha (**A, B**), progestogen receptor (PR) (**C, D**), leukemia inhibitory factor (LIF) (**E, F**), vascular endothelial growth factor (VEGF) (**G, H**), inducible nitric oxide synthase (iNOS) (**I, J**) and endocannabinoid receptor (CB1) (K, L) in the chorionic villi observed by immunohistochemistry. Positive staining was detected in the chorionic villi from both the LNG-EC group (**A, C, E, G, I**) and the control group (**B, D, F, H, J**). The immunostaining for ER-alpha (**A, B**) and PR(**C, D**) was observed in the nuclei of cytotrophobast, syncytiotrophoblast, and stromal cells from both groups. The immunostaining for LIF (**E, F**), VEGF (**G, H**), iNOS (**I, J**) and CB1 (**K, L**) was seen in the cytoplasm of cytotrophobast, syncytiotrophoblast, and stromal cells from both groups. There were no significant differences between the two groups in the expressions of ER-alpha (**A, B**), PR (**C, D**), LIF (**E, F**), VEGF (**G, H**), iNOS (**I, J**) or CB1 (**K, L**). Magnification: 400×.

**Figure 3:**
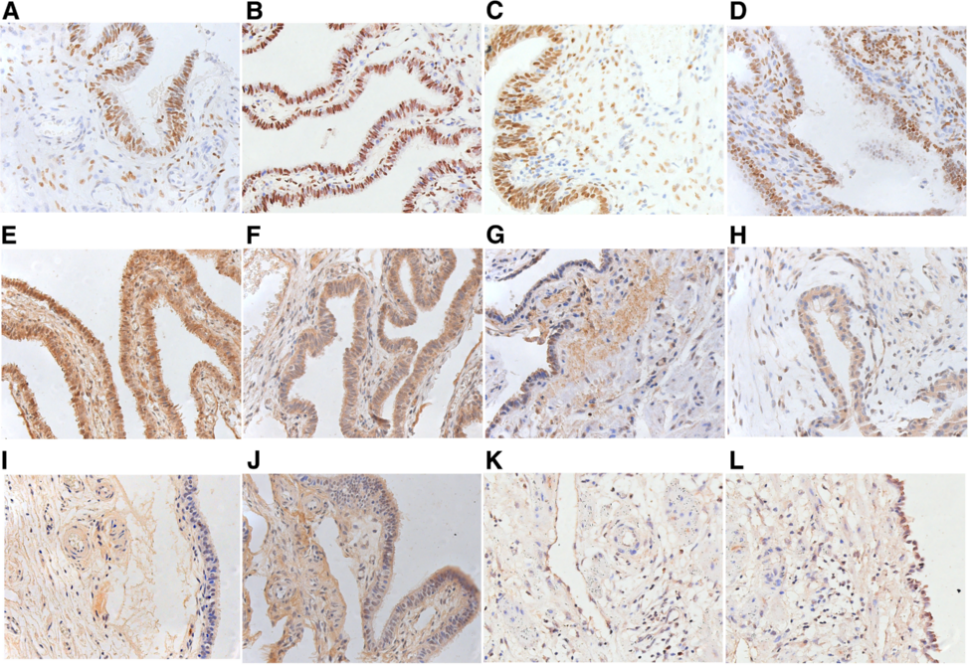
**Immunostaining of the implantation site in the fallopian tube in tubal pregnancy.** Representative images showing immunoreactivity of estrogen receptor (ER)-alpha (**A, B**), progestogen receptor (PR) (**C, D**), leukemia inhibitory factor (LIF) (**E, F**), vascular endothelial growth factor (VEGF) (**G, H**), inducible nitric oxide synthase (iNOS) (**I, J**), and endocannabinoid receptor (CB1) (**K, L**). Positive immunolabeling was detected in the epithelial and stromal cells from both the LNG-EC group (**A, C, E, G, I**) and the control group (**B, D, F, H, J**). The immunostaining for ER-alpha (**A, B**) and PR (**C, D**) was observed in the nuclei of the epithelial and stromal cells from both groups. The positive staining for LIF (**E, F**), VEGF (**G, H**), iNOS (**I, J**), and CB1 (**K, L**) was found in the cytoplasm of the epithelial and stromal cells from both groups. No significant differences were observed in any of the immunoreactivity of ER-alpha (**A, B**), PR (**C, D**), LIF (**E, F**), VEGF (**G, H**), iNOS (**I, J**), or CB1 (**K, L**) between the two groups. Magnification: 400×.

Estrogen receptor-alpha showed a predominantly nuclear presence in both epithelial and stromal cells in the endosalpinx between the two groups. It was also weakly expressed in both nuclei and cytoplasm of the cytotrophoblast (inner layer) and syncytiotrophoblast cells (outer layer) of the trophoblast. The ratios of ER-alpha positively stained ER-alpha in chorionic villi and at the implantation site in the fallopian tube are 3.57+/–1.98 and 3.42+/-0.78, respectively, for the LNG-EC group versus 2.38+/-0.65 and 2.78+/-0.55, respectively, for the control group. However, no significant difference in the immnoreactivity of ER-alpha was detected between the two groups in either chorionic villi (Figure 2A and B) or the tissues at the implantation site in the fallopian tube (Figure 3A and B).

#### Progesterone receptor

PR was expressed at an extremely low level in the nuclei of both cytotrophobast and syncytiotrophoblast cells. There was no significant difference (0.11+/-0.01 versus 0.18+/-0.06, P > 0.05) in the ratio of positive staining for PR in the chorionic villi between the LNG-EC group and the control group (Figure 2C and D). The staining of PR was predominantly observed in the nuclei of both epithelial and stromal cells in the endosalpinx. And no significant difference in the ratio of its positive staining was observed in the tissues at the implantation site of the fallopian tubes between the LNG-EC group and control group (5.02+/-1.14 versus 3.74+/-1.18, P > 0.05) (Figure 3C and D).

#### Leukemia inhibitory factor

The immunostaining for LIF was mainly seen in the cytoplasm of cytotrophobast and syncytiotrophoblast cells, with a positively-stained ratio of 19.98+/-5.12 in the LNG-EC group versus 21.85+/-0.76 in the control group. No significant difference was found in the positive ratio between the two groups (Figure 2E and F). The majority of the epithelial and stromal cells in the endosalpinx were stained for LIF with a positively-stained ratio of 24.29+/-4.70 versus 28.83+/- 2.52 in the LNG-EC group and the control group, respectively. The staining was predominantly in the cytoplasm with a small amount detected in the nuclei as well. There was no significant difference in the LIF immunoreactivity between the two groups (Figure 3E and F).

#### Vascular endothelial growth factor

Immunostaining revealed a predominantly cytoplasmic presence of VEGF in the trophoblast as well as in the epithelial, stromal and vascular endothelial cells of the endosalpinx. The ratio of positive staining in the chorionic villi was 14.76+/-3.16 in the LNG-EC group versus 16.11+/-4.72 in the control group. And at the implantation site in fallopian tube it was 3.26+/-0.98 versus 5.72+/-1.92 (LNG-EC versus control group). No significant changes (P > 0.05) were detected in either chorionic villi (Figure 2G and H) or the tissues at the implantation site in fallopian tube (Figure 3G and H) between the two groups.

#### Inducible nitric oxide synthase

The staining of iNOS was mainly localized in the cytoplasm of both cytotrophoblast and syncytiotrophoblast cells with a small amount also found in the nuclei of the stromal cells in the chorionic villi. The ratio of positively-stained for iNOS in the whole chorionic villi is 4.26+/-1.09 in the LNG-EC group versus 3.91+/-1.61 in control group. At the implantation site of the fallopian tube, the cytoplasm of epithelial cells was positively stained for iNOS with a positive staining ratio of 17.02+/-2.83 in the LNG-EC group versus 12.91+/-2.85 in the control group. Statistical analysis showed no significant difference in the iNOS immunoreactivity in either chorionic villi (Figure 2I and J) or tissues at the implantation site in fallopian tube (Figure 3I and J) between the groups.

#### Endocannabinoid receptor CB1

The trophoblasts as well as the epithelial and stromal cells in the endosalpinx were all positively stained for CB1 in the cytoplasm. Weak staining for CB1 was also detected in the nuclei of the epithelial cells. There was no significant difference in the staining pattern in the chorionic villi between the two groups (positive staining ratio: 2.48+/-0.80 in the LNG-EC group versus 3.58+/-1.39 in the control group) (Figure 2K and L). The positively-stained ratio in tissues at the implantation site in fallopian tube is 5.15+/-1.34 in the LNG-EC group versus 5.28+/-1.21 in the control group, and the difference was not statistically significant (Figure 3K and L).

## Discussion

Levenorgesgtrel-EC acts via inhibiting or delaying ovulation [8,9], but has no demonstrated effect on blastocyst viability or hatching, blastocyst attachment, early implantation, incidence of miscarriage, fetal malformation, pregnancy complications, or any other pregnancy outcome associated with intrauterine pregnancy [28,29]. Up to now, there have been no studies specifically focusing on the molecular changes in either fallopian tube or chorionic villi in tubal pregnancy exposed to LNG-EC.

Same as in intrauterine pregnancy, P, E2 and hCG play crucial roles in ectopic pregnancy. The fallopian tube undergoes decidualization synchronized with the trophoblast development, and that process is regulated by the coordination of P and E2 acting via their receptors [15,28]. Vascular endothelial growth factor and its receptors are known to be involved in the processes of vasculogenesis and angiogenesis in the decidua [17]. An up-regulation of LIF expression promotes decidualization in humans and mice [21]. The iNOS was reported to be present not only in the epithelium, but also in the decidua surrounding the implanted embryos in pregnant mice [22]. The anandamide present in the uterine environment may play an important role in regulating apoptosis through one of its receptors - CB1, and thereby modulate the stability and regression of the decidua during pregnancy [24]. Horne *et al.*[15] reported that ER-alpha was not expressed in the fallopian tube during tubal pregnancy. However, in our study, ER-alpha was expressed in both epithelial and stromal cells of the fallopian tube collected from both groups. This difference might be due to the source of the primary antibody. The presence of ER-alpha in the fallopian tube during tubal pregnancy indicates that E2 might also play a role in tubal pregnancy.

The placenta performs a wide array of metabolic and endocrine functions that are crucial for maintaining normal pregnancy and the fetal development, which are also affected by the balance between P and E2. The expressions of VEGF and its receptors have been found to be correlated with serum beta-hCG concentration, indicating that their expressions may be influenced by the signals sent from the embryo [18]. One interpretation is that increased expressions of VEGF and its receptors promote local angiogenesis, augmenting the supply of oxygen to the embryo [19]. Leukemia inhibitory factor was demonstrated to facilitate blastocyst formation and hatching of human embryos *in vitro*[22]. The iNOS mRNA was detected in both cytotrophoblast and syncytiotrophoblast cells *in vitro*, which suggests that nitric oxide is involved in the placental growth and function [23]. Although the effect of CB1 during placentation is undefined, the endocannabinoid has been shown to be important for successful pregnancy in humans [25]. Based on the findings of the present study, we are tempting to speculate that failure of LNG-EC itself seems to have no effect on the decidualization process or placental development in tubal pregnancy.

We have eventually noticed that the methodological weaknesses in this study could have resulted in serious biases. Another ongoing study implementing a larger sample size is to address the association between the tubal pregnancy exposed to LNG-EC and its clinico-pathological features and outcomes. Furthermore, the pharmacokinetics study of LNG shows that the serum level of hormone is above the threshold from 1 to 6 h after the intake of 1.5 mg LNG and with a half-life ranging from 20 to 60 hours [30]. When the patients were diagnosed tubal pregnancy, LNG-EC has already metabolized. The immediate effect of LNG-EC to the fallopian tube is still undefined. Since the cilia beat frequency of the fallopian tube significantly reduced after the treatment with high doses of progesterone, we hypothesize that the LNG-EC, a temporarily high dose of progestin, can disturb the synthetic balance of P and E2 right after the intake. Due to the ethical limits, it is of paramount importance to conduct LNG study using *in vitro* culture and animal models to cast more light on the association between the LNG-EC and tubal pregnancy.

## Conclusions

In summary, we found that administration of LNG-EC during the cycle of conception had no effect on the expressions of a variety of molecules regulating the pathophysiological changes induced by tubal pregnancy in either chorionic villi or fallopian tube. Therefore, we speculate that the pathophysiological features of tubal pregnancy following LNG-EC failure did not differ from those unexposed to LNG-EC. We propose that a routine therapy for tubal pregnancy can thus be applied to women with tubal pregnancy exposed to LNG-EC and there was no need for additional interventions.

## Abbreviations

EP: Ectopic pregnancy; LNG: Levonorgestrel; EC: Emergency contraception; ER-alpha: Estrogen receptor-alpha; PR: Progesterone receptor; LIF: Leukemia inhibitory factor; VEGF: Vascular endothelial growth factor; iNOS: Inducible nitric oxide synthase; CB1: Endocannabinoid receptor 1.

## Competing interests

The authors declare that they have no competing interests.

## Authors’ contributions

JZ was substantial in charge of study conception and design, data analysis and interpretation, as well as drafting and critically revising the article. CH contributed to data interpretation, drafting and revising the article. MZ, CM, WS and LS were responsible for acquisition of data and drafting part of the article. All authors read and approved the version to be published.
